# Exploring the activities and outcomes of digital teaching and learning of practical skills in higher education for the social and health care professions: a scoping review

**DOI:** 10.1007/s44217-022-00022-x

**Published:** 2023-01-03

**Authors:** Anne Söderlund, Aurelija Blazeviciene, Maria Elvén, Alina Vaskelyte, Raimonds Strods, Inguna Blese, Heikki Paakkonen, Antonio Fernandes, Daniela Cardoso, Sultan Kav, Cigdem Baskici, Camilla Wiktsröm-Grotell

**Affiliations:** 1 Section of Physiotherapy, School of Health, Care and Social Welfare, Mälardalen University, Box 883, 721 23 Västerås, Sweden; 2 Department of Nursing, Lithuanian University of Health Sciences, Kaunas, Lithuania; 3 Centre for Educational Growth, Riga Stradiņš University, Riga, Latvia; 4 School of Business and Health Care, Arcada University of Applied Sciences, Helsinki, Finland; 5 Health Sciences Research Unit: Nursing, Nursing School of Coimbra, Coimbra, Portugal; 6 Faculty of Health Sciences, Başkent University, Ankara, Turkey; 7 Graduate School and Research, Arcada University of Applied Sciences, Helsinki, Finland

**Keywords:** Digital teaching, Digital learning, Digital simulation, Practical skills, Health profession, Higher education

## Abstract

Higher education for health care professionals faces numerous challenges. It is important to develop and apply methods supporting education, especially the practical skills. This scoping review aimed to explore the activities and learning outcomes of digital technology in practical skills teaching and learning in higher education for the social and health professions. Scoping review recommendations and the PRISMA-ScR checklist were applied. Randomized controlled trials published between 2016 and 2021 involving students in higher education who were taking courses in the social sciences and health care and reported interventions with digital technology activities and practices in practical teaching and learning were included. The CINAHL Plus, PubMed, Scopus, ERIC, and Sociological Abstracts/Social Services Abstracts databases were searched. Teaching methods were blended, e-learning or other online-based, and digital simulation-based activities. Teaching and learning environments, methods, resources, and activity characteristics varied, making a summary difficult. Interventions were developed in a face-to-face format prior to digitalization. The outcomes were measured at the knowledge level, not at the performance level. One-third of the studies showed a significant improvement in practical skills in the intervention group in comparison to the control conditions. The use of digital technology in the learning and teaching process have potential to develop of students' skills, knowledge, motivation, and attitudes. The pedagogy of technology use is decisive. The development of new digital methods for teaching and learning practical skills requires the engagement of students and teachers, in addition the researchers.

## Introduction

The higher education of health care professionals has many challenges such as a lack of clinical teachers [1, 2] and opportunities for learning through direct contact with the patients [3], which, in turn, can limit the number of students accepted into the health care programs and thus negatively affect health care resources in the long term. Furthermore, research has shown that students’ perceived stress e.g., fear of making mistakes in clinical placements, is quite high [4]. Thus, it is important to develop and apply methods that can support education, especially the teaching and learning of practical skills.

The term “skills” can mean e.g., clinical examination skills, clinical reasoning skills, and communication skills [5]. Practical skills in health care can include history taking, physical examination and different types of procedural skills, which also require communication skills [6]. The digital teaching and learning of practical skills in higher education for the health care professions can be supportive methods of education. There are many digital teaching and learning modalities, such as online-offline (e.g. where study material, instructions and tasks are delivered online versus offline) [7], e-learning (a type of distance learning) [8], mobile (digital) education, and digital simulation-based [7], as well as blended learning, in which more traditional modalities, such as face-to-face teaching, are integrated with digital recourses [9]. Additionally, the term hybrid learning is often used and referred to as synonymous with blended learning [10].

In a survey concerning dental education in Canada about the use of virtual patients in education, 63% of schools had used virtual patients, and approximately 30% of these had been using virtual patients for more than 10 years [11]. In a study of high-fidelity simulation (defined as high-fidelity manikins, digitally simulated scenarios) in the teaching of nursing students in nursing procedures, both students and teachers were satisfied with the use of the high-fidelity simulation and with the academic outcomes [12]. A flipped classroom model with digital learning as preparation was used instead of peer-based teaching in the context of emergency operation practical teaching for students in human and dental medical training. The students expressed a high level of satisfaction with this model of learning [13]. Digital learning in physiotherapy was studied in a systematic review [8]. The authors concluded that the included studies primarily used blended learning, in which theoretical knowledge was digitally delivered while practical skills training was conducted on campus [8].

The learning environment is important in education [14], and the virtual learning environment (VLE) is equally significant for digital teaching and learning. VLEs can be web-based, providing course-related materials to students. VLEs mostly include assessment, tracking, collaboration, and communication modules that can be accessed by students and teachers regardless of physical location [15]. Furthermore, the framework of a student-centred and competency-based approach to learning, which entails the active engagement of students in the learning process through authentic, meaningful and positive learning experiences [16, 17], is important.

Digital teaching and learning, especially virtual patient simulations, have been studied in many health care disciplines [18, 19], including medicine, dentistry, nursing, and physiotherapy, with mixed evidence of their effects on knowledge [18, 19], clinical reasoning and student satisfaction [18] in comparison to traditional education methods [18, 19]. However, no recent reviews appear to include all kinds of digital teaching and learning methods in the practical skills education of students in various health care professions to provide a more complete picture. Thus, to provide a more complete picture, this scoping review aimed to explore the activities and learning outcomes of digital technology in practical skills teaching and learning in higher education for the social and health professions. The specific review questions were as follows:

- What were the teaching and learning environments, digital teaching methods, and characteristics of the digital teaching activities?
- What were the outcome measures and results of the digital teaching interventions?

## Methods

The recommendations for a scoping review [20, 21] and the PRISMA-ScR checklist in the results reporting [22, 23] were applied. The scoping review was registered in the Open Science Framework, OSF registry (Registration https://doi.org/10.17605/OSF.IO/BVP3M*).*

### Eligibility criteria

The studies needed to meet the following inclusion and exclusion criteria:

Inclusion criteria: Randomized controlled trials (RCTs) and relevant original studies from systematic reviews/meta-analyses, published during the past five years (due to the rapid development and publication rate of digital teaching method studies the five-year study period was chosen in the middle of May 2021, and thus the search was limited to between May 2016 to May 2021) in peer-reviewed journals; studies that involved students in higher education in the field of social and health care; interventions that concerned activities and practices of digital technology in practical teaching and learning; and articles written in English. We decided to include only RCT studies because these still represent the highest scientific level of design and may be more reliable in terms of quality than many other designs.

For Population-Concept-Context descriptions in search terms, see Table 1.

**Table 1:** Key words as Population-Concept-Context, and search history

		Hits
Population		
1.	"teacher*"[Title/Abstract] OR "supervisor*"[Title/Abstract] OR "lecturer*"[Title/Abstract] OR "educator*"[Title/Abstract] OR "Faculty"[Mesh] OR "Teaching"[Majr]	159,396
Concept		
2.	"digital teaching"[Title/Abstract] OR "digital learning"[Title/Abstract] OR "digital education"[Title/Abstract] OR "hybrid teaching"[Title/Abstract] OR "hybrid learning"[Title/Abstract] OR "hybrid education"[Title/Abstract] OR "on-line teaching"[Title/Abstract] OR "online teaching"[Title/Abstract] OR "on-line learning"[Title/Abstract] OR "online learning"[Title/Abstract] OR "online education"[Title/Abstract] OR "on-line education"[Title/Abstract] OR "moodle"[All Fields] OR "e-learning"[All Fields] OR "elearning"[All Fields] OR "Web-based training"[Title/Abstract] OR "digital training"[Title/Abstract] OR "e-tutorials"[All Fields] OR "online tutorials"[Title/Abstract] OR ("e-based"[All Fields] AND "technolog*"[All Fields]) OR "Computer-Assisted Instruction"[Title/Abstract] OR "computerized self-instruction program*"[Title/Abstract] OR "computerized programmed instruction*"[Title/Abstract] OR "e-simulation"[All Fields] OR "esimulation"[All Fields] OR "wiki*"[All Fields] OR "blog*"[All Fields] OR "online quiz*"[Title/Abstract] OR "computer assisted instruction*"[Title/Abstract] OR "virtual reality"[Title/Abstract] OR "educational technology"[Title/Abstract] OR "Instructional Technology"[Title/Abstract] OR "distance learning"[Title/Abstract] OR "distance education"[Title/Abstract] OR "distance teaching"[Title/Abstract] OR "high fidelity mannequin*"[Title/Abstract] OR "blended learning"[Title/Abstract] OR "blended teaching"[Title/Abstract] OR "blended education"[Title/Abstract] OR ("virtual reality"[MeSH Terms] OR "education, distance"[MeSH Terms] OR "Computer-Assisted Instruction"[MeSH Terms] OR "educational technology"[MeSH Terms] OR "Simulation Training"[MeSH Terms])	155,983
3.	"practical skill*"[Title/Abstract] OR "practice skill*"[Title/Abstract] OR "surgical skill*"[Title/Abstract] OR "practical training"[Title/Abstract] OR "professional skills*"[Title/Abstract] OR "Clinical Competence"[MeSH Terms]	102,700
Context		
4.	"higher education"[Title/Abstract] OR "universit*"[Title/Abstract] OR "facult*"[Title/Abstract] OR "education, professional"[MeSH Terms] OR "education, graduate"[MeSH Terms] OR "Universities"[MeSH Terms]	731,402
5.	#1 AND #2 AND #3 AND #4	3922
Limits	Meta-Analysis, Randomized Controlled Trial, Systematic Review, in the past 5 years, Humans, English	289
Total		289

Exclusion criteria were as follows: Studies that were not at the PhD, master’s, or bachelor’s level; studies targeting medical doctors being in “residence” education; being postgraduate education while working at, for example, hospitals; studies targeting medical doctors who were “trainees” in specialist-level education; studies targeting nurses in specialist education that did not include a master’s degree; studies targeting veterinary students; or studies combining residents and students.

### Information sources and search strategy

To identify relevant studies, the CINAHL Plus, PubMed, Scopus, ERIC, and Sociological Abstracts/Social Services Abstracts databases were searched on one occasion on the May 20, 2021. The search was defined as within the previous 5 years (May 2016–May 2021) and was conducted with relevant MeSH search terms and free text terms. The search strategy is shown in Table 1.

The PubMed search resulted in 289 hits; CHINAL Plus, 1 hit, which was already included in the PubMed search results, Sociological Abstracts 0 hits, SCOPUS 19 hits which already were in the PubMed search results; and ERIC, 1 hit, which was already included in the PubMed search results.

### Selection of sources of evidence

The first author, together with a university librarian, conducted the search. In the first step, eligible abstracts (N = 289) were divided among the participating universities (N = 6) and thereafter screened by two researchers from each university (i.e., AS, AB, ME, AV, RS, IB, HP, AF, DC, SK, CB, CW-G). The COVIDENCE system (https://www.covidence.org/) does not reveal the inclusion/exclusion decisions of one screener to the other. If the two researchers came to different conclusions regarding the inclusion/exclusion of the abstract, the decision proceeded to a third person (the first author, who also invited all participating researchers to use the COVIDENCE system), who made the final decision. If the inclusion decision was uncertain, the study was included for the second step, which was full-text reading. The second step was managed in the same manner as the first. The selection of the studies was finally discussed in a whole group meeting (representatives from all six universities) for inclusion agreement.

### Data charting process and parameters

The data from the included studies from each university were tabulated by the respective university researchers. Data for the aim-relevant headings were tabulated (Table 2). The headings were discussed and decided in a whole group meeting. Table 2 includes the following headings: authors, year, country; aim/purpose; participating teachers/educator/developer/delivering person, qualification; target group for teaching, age, gender, level of education; learning environment and education program; digital teaching experimental intervention; comparison intervention; outcome measures; risks and additional comments (in teaching/problems/disadvantages); main findings.

**Table 2:** Characteristics of the 49 included studies (3 dental studies, 30 medical studies, 15 nursing studiets, 1 midwifery study)

Authors, year, country	Aim/Purpose	Participating teachers/educators/developers/delivering person (n =), qualification	Target group for teaching (n =), age (mean), gender, level of education	Learning environment (e.g., platform for education management), and education program	Digital teaching experimental intervention	Comparison intervention	Outcome measures	Risks and additional comments (in teaching/problems/disadvantages)	Main findings
*Dental students*									
Soltanimehr et al. [44], 2019, Iran	In dental students to study the effect of virtual and traditional education on knowledge and skills of interpretation of jaw lesions	The same teacher/mentor (n = 1) taught both groups	N = 39, age (mean = 24 years), fourth year dental students	Nonvirtual environment, dentistry program	6 weeks classes. Virtual learning package included a combination of e.g., quizzes, homework, online weblinks (multimedia contents about radiographic jaw bony lesions), and interactions between students and mentors in the reporting phase	6 weeks classes. Traditional classroom lectures, six one-hour sessions	Theoretical knowledge test with questions and structured clinical examination	The students’ computer skills could have complicated the results	Virtual learning was more effective compared to traditional lecture-based method for increasing theoretical knowledge, but not in clinical skills post intervention. The results were not sustained at the 2 months follow-up
Vincent et al., [39], 2020, France	Virtual reality training compared to analogic training of cavity preparations	Teachers delivered debriefing after training sessions in both groups	N = 88 first-year dental students. No demographic data was reported	Non-virtual environment, dentistry program	Virtual reality training of cavity preparations	Analogic training of cavity preparations	Performance-related parameters (% of tissue removed, drilling time) with the virtual reality simulator and for the control group evaluated by the teachers	Virtual reality-based training allowed a more objective outcome assessment criteria compared to training conducted on plastic analogue teeth	Both groups improved significantly in drilling skills and had similar results on the final exam
Zhang et al. [46], 2020, China	Study the evaluation methods and effectiveness of a virtual simulation platform	Teachers in the dentistry program. The virtual reality system was developed earlier by researchers/university teachers	n = 80, ages 18–21 years, second- and third-year undergraduate stomatology students	Nonvirtual environment, dentistry program	Virtual reality simulation- group (2) Virtual reality system and jaw simulation model- group (3) Jaw simulation model and virtual reality system- group (4) Mandibular molars were created in the virtual simulation system Each student group participated in an eight-hour operating training session	Jaw simulation model of mandibular molars- group (1)	Subjective evaluation of preoperative preparation and intraoperative operation Overall implant accuracy with a scan Students’ satisfaction was measured with a questionnaire	Training time was too short to show long-term effects of the different teaching methods	Group 3 was significantly better in theoretical knowledge compared to other groups. Group 4 was significantly better than the groups 1 sand 2 in knowledge Students’, in the combined jaw simulation model and virtual reality system (groups 3 and 4), implant accuracy skills improved significantly and they were more satisfied with using the combined jaw simulation model and virtual reality system compared to the jaw simulation model group
*Medical students*									
Banaszek D et al. [58], 2017, Canada	To compare arthroscopic skill acquisition in high-fidelity and low-fidelity simulators and to assess skill transfer to a cadaveric specimen, simulating intraoperative conditions	No data	N = 40 surgical novices medical students, age (mean = 23.9 years)	Non-virtual environment, medicine program	High-fidelity simulator: ARTHRO VR virtual reality simulator (GMV), a computer-based system with an integrated arthroscopic curriculum and feedback system Low-fidelity simulator: a bench-top simulator (Sawbones) has a bone model in a simulated soft-tissue sleeve, a decommissioned arthroscopic camera and tactile feedback to the user	No training group	Global Rating Scale (GRS), a 14-point arthroscopic checklist, a timer for procedural efficiency, virtual reality simulator motion analysis	It is not clear if the results could be generalized to older students	Both high-fidelity and low-fidelity simulation trainings were significantly effective in gaining arthroscopic skills. High-fidelity virtual reality simulation was significantly better compared to bench-top simulation in the laboratory and when performed on a cadaver
Basheer et al. [49], 2019, India	To compare accuracy of blood pressure(BP) recorded by students trained on a BP simulator vs. those trained on student colleagues	Staff in faculty of physiology	First-year medical students, n = 145, 53.8% female. No other demographic data was reported	Non-virtual environment, medicine program	Blood pressure simulator	Trained by student colleagues	Accuracy of BP measurement on a structured clinical examination on real patients		There were no differences between the groups The simulation-based training in BP accuracy and methodology might not translate to real life competence
Berger et al. [24], 2019, Germany	To evaluate the effects of a classical cardiopulmonary resuscitation, (CPR) education compared with a bilateral approach to CPR training with problem-based learning (PBL) and high fidelity simulation	Study investigators, medicine doctors	Fourth-year medical students, n = 112, age (mean = 24.5 years), 64.5% female	Non-virtual environment, medical program	30-min-lecture on CPR basics and a 45-min theoretical PBL module followed by 45 min of high fidelity simulated CPR training	Control group participated in a 30-min-traditional lecture on CPR basics and 90-min tutor-guided CPR hands-on-training	The rate of recognizing clinical cardiac arrest followed by sufficiently performed CPR. A follow-up was conducted after 6 months		PBL combined with high fidelity simulation training significantly improved the performance of CPR compared to control condition At six months post instruction, these differences remained only in the hands-off-time, which was lower for the PBL + high fidelity simulation training group
Bommer et al. [64], 2018, USA	Effect of basic orientation to the simulation environment for anxiety, confidence and clinical decision making	University surgical faculty staff, a surgical resident assisted in intervention	Fourth-year medical students, n = 24, age (mean = 28,3 years), male = 16, female = 8	Non-virtual environment, medicine program	First, a two-week surgery intern preparatory curriculum (S-IPC) including instructional and interactive learning Second, the basic orientation was given before the first and second simulations and took five minutes in the simulation scenario environment. It including physical exam findings, the displays in the room, and contact methods for the care team	First, a two-week surgery intern preparatory curriculum (S-IPC) including instructional and interactive learning Second, the basic orientation was given only before the second simulation The simulations in both groups were done with SimMan 3G simulators	Anxiety: Short form of STAI Cato confidence scale Individual learner performance: Adapted NOTECHS	Small sample size No control of previous simulation exposure	The two times oriented students reported significantly lower anxiety and higher confidence and performance scores in all three simulations compared to the group with only one orientation
Brinkmann et al. [25], 2017, Germany	To study which training method leads to better acquisition of basic skills before application of the skills in a simulated surgical procedure	Staff at surgical Skills Lab of university hospital	36 medical students. No demographic data was reported	No information about virtual environment provided, medical program	5-day structured laparoscopy training curriculum with box trainers	5-day structured laparoscopy training curriculum with virtual reality, VR, trainers	GOALS score, observers Global Operative Assessment of Laparocic Skills (GOALS), Self-assessment scale	There were differences between groups before intervention	Both groups (box and VR trainers) improved significantly in laparoscopic basic skills compared to baseline. There were no differences between groups The box-trained group performed significantly better in GOALS score (had higher technical skills)
Buescher et al. [26], 2018, Germany	To investigate the effect of motion parameter feedback on laparoscopic basic skill acquisition and retention during a box training curriculum	Staff at university hospital training centre	Medical students, surgical novices n = 36. No demographic data was reported	No information about virtual environment provided, medical program	5-day training program Lap-X hybrid laparoscopic simulator providing motion parameter feedback and performance comparison during the training	5-day training program of laparoscopicy with regular box trainer	Task performance time, operating time, path length of the left and right instrument, number of movements of the left and right instrument	Small sample size	Both groups improved significantly in their laparoscopic skills The motion feedback group was significantly better in operation time and motion economy when compared to the box training group
Cervantes et al. [65], 2019, USA	To investigate the usefulness of Computer-based video instruction (CBVI) in dermatologic procedure training for medical students	Dermatology faculty	Twenty-nine (n = 29) first- and second-year (25 first- year and 4 s-year) medical students. No demographic data was reported	No information about virtual environment provided, medical program	CBVI-group: Basic dermatology curriculum videos with demonstrations on shave biopsy, punch biopsy, and wound closure sutures A PowerPoint presentation about indications, contraindications, and complications of performing a shave and punch biopsy In-person live demonstration on cadaver tissue	Standard group (SG): A PowerPoint lecture about indications, contraindications, and complications of performing a shave and punch biopsy In-person demonstration on cadaver tissue	Video recorded student performances were graded on a 5-point Likert scale by three dermatologists A multiple-choice cognitive assessment and a survey to capture their laboratory experiences	The retention knowledge test was limited to 1 week, thus, long-term benefit of CBVI and simulation skills are unknown	The CBVI-group scored significantly higher in procedural and knowledge tests in post demonstration and 1 week after
Cheung et al. [59], 2016, Canada	To explore the utility of web-based observational practice (OP) with reading materials (RMs), or OP and collaboration (COP) with reading materials or reading materials (RM) only to prepare novice medical students for simulation-based mastery learning (SBML) in central venous catheterization (CVC)	An expert in simulation	Undergraduate medical students (n = 30) without previous experience in CVC. No demographic data was reported	Web based learning, medical program	Two intervention groups: a web-based learning with an instructional video demonstrating CVC on a simulator by an expert and a video-based “ spot-the-difference” activity + reading materials (OP-group); a collaborative OP activities + reading material (COP- group)	Reading material (RM-group)	Time to completion and retention test performance with a task-specific checklist and a global-rating scale (GRS)	The OP and COP groups had more instructional materials to prepare for the SBML workshop, and this, might have been a confounder	The OP and COP-groups took significantly less time in completing the SBML workshop compared to the RM-group. No differences at one week follow-up
Chidambaram et al. [31], 2019, UK	To assess the transferability of knowledge of cognitive task simulation and rehearsal app, on the learner’s ability to perform laparoscopic cholecystectomy	Staff at Kinosis Limited, London, UK	Medical students (n = 40) in their medical, second to fifth year, age: 21.9 ± 1.3; 21.4 ± 1.3), females 21, males 19	No information about virtual environment provided, medicine program	The Touch Surgery mobile program is an interactive application that uses Cognitive task analysis (CTA) framework and multimedia animations to teach the operative steps of procedures and specialties Preparation: A 10-min introduction to laparoscopic equipment and a 15-min educational tutorial on laparoscopic cholecystectomies	Written information about intraoperative steps and the order in which they are performed Time studying the material was limited to 15 min per student	A cognitive performance scale was used	The baseline performance was not measured No long-term retention was measured	The Touch Surgery group showed a significantly higher total mean performance score for laparoscopic cholecystectomies compared to the written information group
Coret et al. [61], 2018, Canada	To introduce medical students to the intellectual and developmental disabilities (IDD) population by using a blended education including video narratives of and interactions with people affected by IDD	Staff at faculty of McMaster University	27 first-year medical students were randomized into either control (n = 12) or narrative intervention (n = 15) groups, female:23 male: 4, age (mean = = 23.1 years)	A blended educational approach, no specific information on digital environment, medical program	Narrative intervention group: Introductory video lecture about IDD healthcare. Two additional videos on “Communicate CARE: Caring for People with Developmental Disabilities Primer” and a discussion of videos about people living with IDD and their perspectives and 4 simulated clinical encounters with patient educators (PEs) who have lived experiences of IDD	Control group: Introductory video lecture about IDD healthcare and a quiz and a discussion on correct responses and 4 simulated clinical encounters with patient educators (PEs) who have lived experiences of IDD	A quiz, communication skills assessment, attitudes assessment students’ performance in the clinical encounters, along and self-reports of comfort, confidence, and competence of interacting with people with IDD	The sample size was small and the communication skills rating forms were not clearly worded Long-term impact was not measured	The Narrative and control groups did not differ significantly in communication assessments nor did they differ in self-reported comfort, confidence, and competence when interacting with people with IDD
Drummond et al. [37], 2017, France	To compare an online course and a serious game as pretraining before learning the management of sudden cardiac arrest	Staff in the department of Simulation in Healthcare A pediatric, Anesthesiologist and nurse anesthetist (n = 3) rated participants actions; qualified instructors in simulation	Eighty-two (n = 82) second-year medical students from two medical faculties, age (mean = 21 years), male = 26, female = 56	Non-virtual environment, medical program	The serious game used was Staying Alive, including a 3D realistic environment Participants pretrained on day 1 and day 7 with the Staying Alive game or online course before simulation-based management of cardiac arrest. On day 8, each participant was evaluated repeatedly on a scenario of cardiac arrest until reaching a minimum passing score	An online course with a PowerPoint lecture	Total training time needed to reach the minimum passing score on day 8 This was also assessed 4 months later	Results may not be generalizable to real-life conditions	The serious game used in this study was not significantly better than the online course to pretrain medical students in the management of a cardiac arrest
Drummond et al., [38], 2018, France	To compare two pedagogical approaches training students once on three different scenarios of paediatric asthma exacerbations (PAEs) vs. training students three times with the same scenarios of PAE	Staff at department of Simulation in Healthcare	N = 85 third-year medical students, novice learners for the management of PAEs, male = 29, female = 56, age (mean = 22 years)	Non-virtual environment, medical program	The high-fidelity manikin SimBaby (Laerdal, Stavanger, Norway) was used only once in the three different PAE scenarios	The high-fidelity manikin SimBaby (Laerdal, Stavanger, Norway) was used three times on the different PAE scenarios	A checklist-based performance score on a scenario of PAE was used The assessments were conducted at 1 week and 4 months after the training, on a completely new PAE scenario	Study included volunteer students and the generalizability is questionable	There were no differences between groups at any of the assessment occasions
Elledge et al. [32], 2018, United Kingdom	To evaluate the “flipped classroom” in a single module on maxillofacial radiology in a course on trauma skills	No data	Medical students (n = 29). No demographic data was reported	Virtual learning environment, no further specification, medical program	The e-learning course was created with Articulate360 and the tool Rise, and was shared with the participants via a weblink that they could use as often they wanted before the course In the face-to-face course, an interactive tutorial was given based on the problems and learning objectives they had identified	Traditional lecture during the face-to-face course	Knowledge and confidence were measured with 20 questions with single-best-answers, and 10 situations to be assessed with a visual analogue scale before and after the face-to-face course	The sample size was small It was not possible to monitor how many students accessed the online resource and how often	There were no significant differences between groups for any of the measures. Both groups improved significantly in the 10 situations assessment but not in the 20 questions assessment
Egro et al. [66], 2018, USA & United Kingdom	To provide evidence supporting the role of an e-learning, (EL) on acute burns management (“basic burns management”/ BBM) when compared with traditional teaching(TL) methods	A tutor was present during the interventions	N = 79 medical students of varying years of education. No demographic data was reported	A 33-webpage EL tool (www.basicburnsmanage-ment.com) was created A 10-min video was also developed to aid students to demonstrate how to approach burn patients in an emergency setting and reflect on what they have learned	Basic Burns Management e-Learning: 1-h self-directed learning A tutor was present during the EL session for assistance and to answer questions	A traditional lecture one-hour teaching session covering the same topics as the Basic Burns Management EL tool. The lecture was presented by a tutor	Structured exam with 10 knowledge-related questions Student satisfaction with the teaching modality was measured by 11 questions Pre- and post-teaching scores were collected	There is a need for long-term follow-up in knowledge retention to validate the teaching methods	Both groups significantly increased their knowledge on the topic. There were no significant differences between groups in knowledge or the satisfaction with teaching module scores
Fransen et al. [41], 2018, Netherlands	To understand of how an e-learning program affects the acquisition of dermatological knowledge and the underlying learning processes of medical students	Staff in the department of dermatology at the university hospital	Fourth-year medical students (n = 62). No demographic data was reported	E-learning program and classroom lectures, medicine program	e-Learning by a smartphone app Education in Dermatology (ED). The interactive web-based program contains 35 clinical cases, on the common dermatological diseases. Each case consists of two or three multiple-choice questions regarding the description, diagnosis, and management of the disease	Conventional teaching consisted of one lecture addressing dermatological topics	Pre- and post-intervention knowledge tests where the questions were based on the learning objectives and outcomes of the medical curriculum Qualitative interviews of each individual to explore learning approaches	Only the short-term retention of knowledge was measured	The e-learning program group had a significantly higher improvement in dermatological knowledge compared to the conventional teaching group The qualitative data indicated that the process of learning was more efficient in the e-learning group
Hempel et al. [27], 2016, Germany	To assess whether pre-course e-learning based on clinical cases in the form of screencasts was equivalent to classroom-based lectures in preparation for hands-on training during ultrasound courses	Physicians, all certified instructors and experienced in e-learning program (n = 7) All trainers participating in the hands-on training were experienced medical educators	Medical students (n = 62), 37 women, 25 men, third year and more, age (mean = 23)	E-learning system and classroom-based, medical program	Group 1 (G1) (n = 29) had a pre-course e-learning, with 14 short screencasts, discussion, and a standardized hands-on training session	Group 2 (G2) (n = 31) received classroom-based presentations on the day of the course before an identical hands-on training session as G1	Multiple-choice knowledge pre-course test, a practical structured clinical exam post-course test. Multiple-choice test after the hands-on training session and 1 day after the course	The flexibility in space, time, and pace are advantages of e-learning but the disadvantage is lack of learner–teacher interaction	G1, after the e-learning, performed significantly better on the pre-course test compared with G2 No significant differences between groups in the clinical exam or the knowledge tests post course
Linsk et al. [70], 2018, USA	To evaluate the VBLaST-PC ^©^, training compared with the Fundamentals of Laparoscopic Surgery training	No data	N = 24 medical students. No demographic data was reported	The VBLaST-PC system, medicine program	Fundamentals of Laparoscopic Surgery (FLS) training group, Or a Virtual Basic Laparoscopic Skill Trainer (VBLaST-PC ^©^) training group Fifteen training sessions, 30 min in duration per session per day, were conducted over 3 weeks	No training period before using either of the simulators	Performance data, including time, error, FLS score, learning rate, learning plateau, and cumulative summation, CUSUM, score, were analysed All subjects completed pre-test, post-test, and retention test (2 weeks after post-test)	Very small sample size	All trained subjects showed significantly better performance in FLS. No significant differences between the three groups in FLS The VBLaST results were similar
Liu C et al. [56], 2016, Australia	This study aimed to evaluate the effectiveness of EQClinic to improve the clinical communication skills of medical students	A trained tutor was present in the room to observe and assess the performance of the student during the consultation with the Standardized patient, SP	N = 268 s-year medical students, n = 108 male and n = 160 female	Nonvirtual environment, medical program	To complete online communication skills EQClinic training during weeks 1–5 (group A) EQClinic has an automated visual presentation of students' nonverbal behaviour coupled with human feedback from a standardized patient (SP)	Group B was control group during the weeks 1–5 Group B completed online communication skills EQClinic training during weeks 8–11	Student-Patient Observed Communication Assessment (SOCA) was used by evaluators at 2 time points and by SPs (n = 83) in face-to-face consultations	Consultations were limited to a history taking. A low proportion of students (30%) completed all components of the intervention	Group A’s SOCA communication scores were significantly higher compared to group B at post-intervention After group B had had the intervention there were no significant differences between groups in SOCA
Kron FW et al. [69], 2017, USA	To assess advanced communication skills among second-year medical students participating in a computer simulation (MPathic-VR) on virtual humans or a multimedia computer- based learning (CBL) module	There is no information on the involvement of teachers. MPathic-VR and the CBL module were self-paced	Second-year medical students (n = 421), age (mean = 25,5 years), n = 198 female	Non-virtual environment, medicine program	MPathic-VR was used for teaching advanced communication skills MPathic-VR intervention assumed the role of an intern in two thematically linked scenarios; intercultural (patient-student) and interprofessional (nurse-student) communication	The computer-based learning (CBL) module was used in the control group. This was an open ware “Introduction to Standardized Communication for Health Professionals” program including principles about interprofessional communications and patient scenarios on ineffective and effective communication between doctors and nurses	Objective structured clinical exam om communication skills (OSCE) Communication scores during interactions with MPathic-VR's intercultural and interprofessional communication scenarios (only MPathic-VR- group)		MPathic-VR-group had significantly higher scores on OSCE compared to students in the CBL group MPathic-VR group improved between scenarios in the interprofessional and intercultural communication
Kiesewetter et al. [28], 2020, Germany	To understand how case format affects clinical reasoning outcomes and cognitive load	The cases were written with three experienced physicians	N = 142 medical students (third to sixth year), age (mean = 24.4), 72% female	CASUS (http://www.casus.net). Learners adopted general practitioner role and diagnosed eight VPs, medical program	Virtual patients whole case format	Virtual patients in serial cue format	Diagnostic accuracy, knowledge, and cognitive load		Knowledge and diagnostic accuracy did not differ between serial cue and whole case virtual patient presentations Those with low prior knowledge showed higher cognitive load compared to students with high prior knowledge
Onishi et al. [57], 2019, Japan	To evaluate the proficiency of medical students in acquiring laparoscopic suturing and endoscopic surgical skills in small spaces, using the novel paediatric fundoplication model	An expert paediatric surgeon in open and laparoscopic surgeries	Sixth-year medical school students with no previous experience in training to perform laparoscopic suturing (n = 17). No demographic data available	No information about virtual environment provided, medical program	Virtual group received the suturing instruction using an Infant Laparoscopic Fundoplication Virtual Simulator training—LapVR (CAE Healthcare, Sarasota, FL) The students in each group practised laparoscopic suturing and tying skills for 1 h	Teaching group received the training from an expert in person, who advised the participants as needed The video group received training while watching a training video by an expert laparoscopic paediatric surgeon A box trainer (Endowork Pro II) was used in the video group and the teaching group	Time required to complete the task Suturing balance of the right and left side Suturing intervals The total path length of the forceps with a TrackSTAR system Average velocity of the forceps tips Average acceleration values of the forceps tips Number of tissue injuries	Small number of participants Most of students felt that the fundoplication task was much more difficult than the knot-tying training using the dry-box trainer because of the small working space	There were no significant differences between groups in 6 of the 7 outcomes measures. The average acceleration of the forceps in the virtual group was faster compared to the other groups
Plackett et al. [33], 2020, UK	To assess the feasibility, acceptability and potential effects of an online patient simulations (OPS) eCrest (electronic Clinical Reasoning Educational Simulation Tool) to support teaching of reasoning skills	No information about who developed or delivered the intervention However, real patients contributed to the development of the scripts and identification of clinical and behavioural characteristics for the simulated cases	Final year undergraduate students (n = 264), > 50% were between 23–24 years old and male	No information about virtual environment provided, medicine program	Intervention group; Online patient simulations (OPS) eCREST with three videos of patients (played by actors) presenting to their primary care physician with respiratory problems that could be indicative of serious conditions. The student collects information from the patient, while continually being prompted to review their differential diagnosis After each case they should make a final differential diagnosis and receive feedback	Control group had teaching as usual	The Clinical reasoning with the Flexibility in Thinking (FIT) scale of the Diagnostic Thinking Inventory (DTI) Observed proportion of central questions and examinations Counting the number of times participants changed their diagnosis The selection of the most important diagnosis Relevant medical knowledge with 12 multiple choice questions Feasibility in assessing student uptake Acceptability by retention rates and a survey that consisted of statements on the perceptions of eCREST	Relatively low uptake (18%) The data for clinical reasoning was not collected at baseline	The intervention group identified significantly more important information than the control group The clinical reasoning outcomes showed that the intervention group reduced the effects of the unpacking principle, confirming and anchoring biases There were no significant differences between the intervention and control groups in feasibility and acceptability
Plana et al. [71], 2019, U.S.A	To compare digital simulation to a surgical textbook for conceptualization of cleft lip repair	Two craniofacial surgeons, a philanthropic organization and a biotechnology company All content was developed by craniofacial surgeons in cleft lip and palate care	First-year medical students, volunteer and novice learners (n = 35) No demographic data available	No information about virtual environment provided, medical program	Virtual surgical simulator online group for cleft surgery demonstrating the markings for the extended Mohler unilateral cleft lip repair All participants (both intervention and control group) were given 10 min to draw the preintervention markings for surgical repair, plus 20 min to review their respective educational resources. The participants were given a patient photograph and 10 min to draw surgical markings for a complete unilateral cleft lip repair (postintervention markings)	Textbook group read a chapter describing the detailed markings for the same cleft lip repair technique	Expert scoring of the drawings on two occasions Student satisfaction with the student evaluation of educational quality on two occasions	The grading scale for evaluation of the drawings is not validated	Virtual surgical simulator online group was significantly better in their drawing of the cleft lip repair compared with the textbook group Postintervention scores for all participants were significantly improved compared to the pretest drawing
Poulsen et al. [35], 2019, Denmark	To assess the effectiveness of e-learning combined with simulation-based training or e-learning alone or no intervention in theoretical knowledge and confidence in future patient encounters	No data	Medical students in their last semester of a master’s degree in Medicine (n = 217) No demographic data available	No information about virtual environment provided, medical program	Intervention group 1: education via an interactive case-based e-learning program to be completed at home Intervention group 2: combination of the e- learning program and simulation-based training. The same intervention as e-learning group alone with simulation- based training at Corporate HR, MidtSim	Control group: teaching as usual	Multiple choice questionnaire (MCQ) for theoretical knowledge of pain management, before and after the intervention Level of confidence in management of acute pain	-	The improvement in MCQ test was significantly different better in the intervention groups compared to control condition. There were no differences in MCQ between the two intervention groups The combined e-learning and simulation-based training group had highest confidence level for future acute pain management
Schoeb et al. [29], 2020, Germany	To evaluate a step-by-step mixed reality (MR) guidance system to train medical students to perform bladder catheter placement	A certified expert with a master’s degree in medical education developed this MR system’s pedagogical concept Experienced nursing instructors in the urology department	Medical students enrolled between their fourth and fifth year in a medical doctor program (n = 164), n = 95 female, n = 69 male, Age (mean = 25.2)	No information about virtual environment provided, medical program	Mixed reality guidance system (MR) group: Instructions displayed through a head-mounted display (HMD) and provided step-by-step instructions on how to prepare the materials in a sterile manner, followed by guidance through the placement process. The instructions were provided on demand Hands on training during 30-min on a male catheterization-training model	Control group: Instructions given by an instructor. Hands on training during 30-min	Learning outcomes with OSCE (Objective structured clinical examination) Teaching preference and learning experiences with a questionnaire Self-evaluation of bladder catheter placement Usability of the MR by System usability scale (SUS) and NASA Task load index	Participants often needed help in handling the MR-device and there were technical problems	Learning outcome average scores were significantly better for MR group Teaching and learning experience were significantly more favourable in the control group Self-evaluation of the bladder catheter placement showed no significant group differences The MR system was perceived difficult to use
Shim et al. [48], 2018, South Korea	To compare education methods for skills in robotic surgery using a robotic virtual reality simulator (RVRS)	No data	Medical students, (n = 45), n = 13 females and n = 32 males, no data for age	No information about virtual environment provided, medical program	Intervention group: A robotic virtual reality simulator (RVRS; dV-TrainerTM, Mimic Technologies) taught directly by an expert who demonstrated the task and provided specific feedback for improvement All students had a mini lecture, and watched a video clip, and then viewed a demonstration presented by the same proctor. Warm-up exercises to familiarize participants with the robotic system that were repeated more than 80 times for 60 min every day to obtain a learning curve	Control group 1: educational video to be viewed whenever students wanted Control Group 2: independent training	For the measure of proficiency level the student’s performance was recorded and analysed with a scoring algorithm The measure of learning curve was total task time, economy of motion, master workspace range, and number of instrument collisions and critical errors	The sample size in each group was relatively small, thus compromising the external validity of the results	The mean time for completing the task was significantly better in the intervention group compared to the independent training group as well as the educational video group compared to the independent training group
Schmitz et al. [40], 2020, Switzerland	To examine whether or not the desired effect of hints depends on examples being video-based or text-based	No data	Fourth-year medical students (n = 147), n = 90 female and n = 57 male, age between 19–40 years	No information about virtual environment provided, medical program	Group 1:Video example with hints had access to learning module including video example with hints Group 2:Video example without hints had access to learning module including video example without hints The participants of all groups received online access to an e-learning module to individually prepare for the BBN task. It introduced the SPIKES protocol for delivering bad news and provided the exact same instructions and content across groups. Each hints group was presented with eight identical hints implying the SPIKES steps and their underlying principles	Group 3:Text example with hints had access to learning module including text example with hints Group 4:Text example without hints had access to learning module including text example without hints	The five-point SPIKES scale and the ‘global Breaking Bad News Assessment Scale’ (glBAS)		A significant main effect of hints on both, the SPIKES and glBAS scores’ showed that students learning from the examples with hints were more effectively prepared to deliver bad news compared to those learning from the examples without hints
Stephan et al. [30], 2018, Germany	To study the effect of peers teaching lessons compared to a paediatric basic life support video demonstration	The experimental intervention (video) was developed by the same instructor (n = 1) who was the peer teacher in the control condition	N = 88 fourth year medical students. No demographic data was reported	Non-virtual environment, medical program	Paediatric basic life support video lesson according to European Resuscitation Council guidelines and was recorded by the same instructor who delivered the peer teaching	Experienced peer teaching on an infant manikin	Structured 12-item clinical examination		The peer teacher group showed significantly better results in the structured clinical examination immediately after the course and at the end of the semester compared to the video group. The peer teacher group also had better resuscitation performance
Sugand et al [34], 2019, United Kingdom	To study the effect of number of training sessions with an augmented reality fluoroscopic simulator	There was no information of any engaged teachers in this study	N = 45, age (mean = 21), undergraduate medical students	Non-virtual environment, medical program	Augmented reality fluoroscopic simulator, 10 sessions	Augmented reality fluoroscopic simulator, 2 sessions	Procedural accuracy and time, number of radiographs, number of guidewire retries		The 10 sessions-group showed better improvement in procedural time, number of radiographs, number of guidewire retries compared to 2 session-group
Wu et al. [62], 2017, Canada	To study differences in teaching methods for the diagnosis of ear pathologies	The instructor in both experimental groups was the first author The standard classroom instruction by an otolaryngologist	N = 54, first- and second- year medical students. No demographic data was reported	Non-virtual environment, medical program	Otoscopy simulation or web-based online module for the diagnosis of ear pathologies	Standard classroom instruction	Diagnostic accuracy and otoscopy skills		Postintervention otoscopy simulation and web-based online module groups showed significantly higher diagnostic accuracy. At 3 months follow up the otoscopy simulation group showed greatest improvement both in accuracy and otoscopy skills
**Nursing students**									
Aebersold et al. [63], 2018, USA	To study an anatomy augmented procedure training video with interactive virtual simulation exercises to determine the impact on naso-gastric tube (NGT) placement skills	No data	Sophomore and junior nursing students (n = 69) No demographic data was reported	No information about virtual environment provided, nursing program	iPad anatomy-augmented virtual simulation training The students watched a training video which was followed by interactive virtual simulation exercises	A training video and didactic content in accordance with the standard curriculum. The content was similar in both control and intervention conditions	Competence in placing the NGT with a 17-item competency checklist, satisfaction with the AR technology, and perceptions of AR as a potential teaching tool for clinical skills training	A small sample size	The augmented virtual simulation training group was significantly better in correctly placing the NGT compared to the control group
Akalin et al. [50], 2020, Turkey	To investigate the impact of high-fidelity simulation on nursing students’ knowledge, critical thinking, and clinical decision-making in the management of pre-eclampsia	No data	Third-year nursing students, N = 107, age (mean = 20.8 years), 87% female	No information about virtual environment provided, nursing program	Classroom training (four hours course including video, pictures, PowerPoint presentation, etc.) and high fidelity simulation training	Classroom training (same as in the simulations group)	Knowledge Assessment Form (KAF), California Critical Thinking Disposition Inventory (CCTDI), Clinical Decision Making in Nursing Scale (CDMNS)		The group with high fidelity simulation combined with classical training significantly increased students’ acquisition and retention of knowledge, critical-thinking, and clinical decision making on preeclampsia compared to classical training at least in the short-term
Aloush [55], 2019, Jordan	To assess student nurses’ knowledge of central line–associated bloodstream infection–prevention guidelines (CLABSI) and to compare the effectiveness of simulation versus classroom lecturing	Principal investigator	Fourth-year nursing students, N = 107, age (mean = 2 years), 55% female	No information about virtual environment provided, nursing program	Simulation course	Classroom lecturing	A questionnaire with 23 multiple choice questions specifically developed for the study		Both groups showed significant improvement at post-test in knowledge about CLABSI-prevention guidelines. There was no significant difference in the overall knowledge score between the two groups
Blanie et al. [36], 2020, France	To compare the educational value of simulation by gaming (SG) and a traditional teaching (TT) method to improve clinical reasoning (CR) skills necessary to detect patient deterioration	Nursing instructors	The second- year nursing students, n = 146, age (mean = 25.5 years), 124 female	Nonvirtual environment, nursing program	Simulation by gaming group where the students played individually with a serious game including two cases followed by a debriefing with an instructor	Traditional teaching group working on the same cases as SG in text and a traditional teaching course with a PowerPoint presentation by an instructor	Clinical reasoning was measured with script concordance tests. Students’ global satisfaction, motivation and professional impact were also included Post-intervention and one month after the post-test	Not reported	No significant difference was shown in clinical reasoning to detect patient deterioration or in professional impact between SG and TT groups in any of the timepoints. Global satisfaction and motivation were significantly higher the SG group
Breen et al. [43], 2019, Ireland	To study whether the addition of a proficiency-based progression, PBP, simulation training program in the national NEWS learning module resulted in better performance of clinical communication of a deteriorating patient than the E-learning module alone or in combination with standard simulation	Experienced clinicians and educators who had undergone the Train the trainer NEWS program	Third year nursing students n = 45 and final-year medical students n = 45, age varied between 18–30 + , male n = 17, female n = 73	The Irish health service’s National Early Warning Score, NEWS, e-learning module, nursing program	Group E: The National Early Warning NEWS e-learning only Group E + S: the national e-learning programme plus standard simulation	Group E + PBP:, the national e-learning plus proficiency-based progression, PBP, simulation	Case specific performance assessment based on the Identification, Situation, Background, Assessment, Recommendation communication tool, ISBAR, which incorporated within the NEWS program	Blended sample of nursing and medical students The results were not reported separately for nursing and medical students	Group E + PBP performed significantly better in the ISBAR compared to the other two groups, i.e., they had better ability to reach the proficiency benchmark
Cobbett et al. [60], 2016, Canada	To compare the effectiveness of two maternal newborn clinical simulation scenarios, virtual clinical Simulation, and face-to-face high fidelity manikin simulation	Research assistant, lab instructor	N = 56 third year nursing students, age (mean = 25 years), 84% female	Non-virtual environment, nursing program	Group 1 received the experimental intervention vSim® for Nursing, VCS, for scenario one, preeclampsia, and the F2F high-fidelity manikin simulation for scenario two the Streptococcus, GBS	Group 2 received face-to-face high fidelity manikin simulation intervention for the preeclampsia scenario and the experimental intervention VCS for the GBS scenario	Nursing Anxiety and Self-Confidence with Clinical Decision Making Scale (NASC-CDM). Multiple choice questions for preeclampsia knowledge, and Simulation Completion Questionnaire	A small sample size	Simulation, regardless of approach, did not have a significant effect on knowledge about caring in preeclampsia or GBS Students’ anxiety was significantly higher after using the VCS compared to use of the F2F There was no significant difference. on students' self-confidence after any of the two simulations
Erlinger et al. [67], USA	to compare the use of high-fidelity mannequin-based simulation versus virtual simulation for recognition of intraoperative myocardial infarction (MI)	University’s simulation centre Research team	N = 39 students in their second and third year of a master’s degree program in nurse anaesthesia	No information about virtual environment provided, nursing program	High-fidelity manikin simulation followed by virtual simulation	Virtual Simulation followed by high-fidelity manikin simulation	Length of the time to recognize that an MI or other intraoperative critical event was occurring in both the virtual and the high-fidelity manikin simulations	The sample size was small	Second-year students performed better when using high-fidelity manikin simulation compared to the virtual simulation For the third-year students there was no significant difference between the groups
Franklin et al. [68], 2020, USA	To determine best practices for multiple-patient simulation (MPS) preparation and frequency to improve behavioural performance in priority setting, delegation, and multitasking	Research Team	Nursing students (n = 73) in capstone clinical courses in baccalaureate programs, age varied between 18–44 years	Learning management system to deliver all materials. No other description of the environment, nursing program	Group 1: Expert Modelling, EM, videos that were embedded in VOPP lectures on students' competence and self-efficacy for providing care to multiple patients in simulation lab Group 2: Voice-over power point (VOPP) on students' competence and self-efficacy for providing care to multiple patients in simulation lab	Group 3: Reading assignments regarding articles, policies, and procedures All groups performed pre- and post-intervention the Multiple-Patient Scenario that includes care of three simulated manikin patients at the beginning of the shift	The Creighton Simulation Evaluation Instrument (CSEI) was used to measure competence National League for Nursing Self-Confidence for Learning in Simulation survey was used to measure self-efficacy	The EM and reading groups had more work experience possibly contributing to their competence	No difference in competence scores between groups. There was a significant difference in pre-test and post-test competence scores in all groups. No significant difference in self-efficacy between the groups
Günay İsmailoğlu e al [52], 2018, Turkey	To compare the effectiveness of a virtual intravenous simulator with a plastic arm for teaching intravenous catheter insertion skills	An instructor	N = 65 nursing students, female = 56, male = 9, age (mean = 20.4 years)	No information about virtual environment provided, nursing program	Virtual Intravenous Simulator, VIS, is a VR simulator that enables learning and practising with or without IV catheter insertion related psychomotor skills	The Life/form Adult Venepuncture and Injection Training Arm model is an adult-sized plastic arm with a multivascular system designed for IV injection training	Intravenous catheterization knowledge assessment form, Intravenous Catheterization Skill Test, Self-Confidence and Satisfaction Scale, and Fear Symptoms Scale	Not all participants had performed IV catheter interventions in the clinic. Thus, the effect on practice was not fully evaluated	There were no significant differences between groups in IV catheterization knowledge, IV skills and self-confidence scores at posttest The virtual simulator group was significantly more satisfied with the training method and experienced lower levels of fear symptoms compared to the control group
Lee, NJ et al. [47], 2016, South Korea	To identify the effects of a mobile-based video clip on learning motivation, nursing competency, and class satisfaction	Four evaluators were involved in assessing the students’ performance skills	N = 71 s year nursing students, age (mean = 20 years), 64 female and 7 male	No information about virtual environment provided, nursing program	A female urinary catheterization video clip was created using a female catheterization simulator M180. A voice narration was included with each scene. The total duration was 6 min 30 s. The video clip could be downloaded to all mobile devices, and every 2 days the students were reminded to access the video clip The students also participated on the same a 90-min classroom lecture on urinary catheterization as the control group	A 90-min lecture on urinary catheterization also included the video created for the experimental group	The learning motivation and confidence was measured by the Instructional Materials Motivation Survey (IMMS); urinary catheterization knowledge with 15 questions; the skill performance with a procedure checklist; and self-reported scale for the level of class satisfaction	Students were instructed not to share the video clip with other students, but all students attended the same institution and practised in the same laboratory, so contamination may have occurred	There were no differences between groups in skill performance or knowledge. The intervention group showed significantly higher learning motivation and class satisfaction than the control group. The intervention group was significantly more confident in practising catheterization than the control group
Padilha et al. [42], 2019, Portugal	To evaluate the effect of clinical virtual simulation with regard to knowledge retention, clinical reasoning, self-efficacy, and satisfaction with the learning experience	No data	N = 42 nursing students from the second year, age (mean = 19.9 years), 40 female	No information about virtual environment provided, nursing program	The clinical virtual simulator (Body Interact) presenting virtual patients on a case-based physiological algorithm that recreates a dynamic health conditions that responds to user interventions	The same case-based learning approach as in the intervention group, with recourse to a low-fidelity simulator and a realistic environment	Knowledge and clinical reasoning pre- and post-intervention and 2 months later, with a multiple-choice knowledge test. The students’ levels of learning satisfaction and self-efficacy were assessed with a Likert scale	The follow-up time was too short to fully evaluate the knowledge retention	The experimental group significantly improved in knowledge after the intervention and 2 months later and showed higher levels of learning satisfaction compared to the control condition. There were no significant differences in self-efficacy perceptions
Rossler et al. [72], 2019, USA	To study the effectiveness of the Virtual Electrosurgery Skill Trainer (VEST©) on operating room (OR) fire safety skills	No data	Prelicensure nursing students, n = 26, 22 female. No other demographic data was reported	No information about virtual environment provided, nursing program	OR fire safety education consisting of classroom lecture, presentation of the protocols for fire safety, and interactive clinical scenario discussions focused on the fire triangle and OR team member roles as well as simulation with VEST© module The intervention group completed a training session to orient to VEST© followed by 5 independent and monitored VEST© module education sessions	OR fire safety education consisting of classroom lecture, presentation of the protocols for fire safety, and interactive clinical scenario discussions focused on the fire triangle and OR team member roles	Fire Safety Evaluation test Perioperative Performance Evaluation Tool for Nursing	The sample size was small	Fire Safety Evaluation exam scores showed that both the control and intervention groups were similar in knowledge of fire safety No significant differences were found in knowledge between the groups
Tuzer et al. [53], 2016, Turkey	To study the effects of teaching thorax-, lung- and cardiac examinations with a high-fidelity simulator compared to standardized patients	University simulation laboratory. One teacher (a study investigator) gave a lecture before training. The investigator supervised the training sessions	N = 52, age (mean = 23), fourth-year nursing students. No other demographic data was reported	Non-virtual environment, nursing program	Thorax-, lung- and cardiac examinations with a high-fidelity simulator	Standardized patients	Evaluation of the level of knowledge on thorax, lung, and cardiac examination, Skills assessment form	Small sample size	Performance score differences were non-significant between the groups after the training. Knowledge scores were significantly higher in the standardized patient group
Vural Doğru et al. [54], 2020, Turkey	To study differences between high-fidelity simulator cardiac auscultation training and traditional teaching method	University simulation laboratory or occupational skills laboratory. The researcher was the supervisor in the experimental condition	N = 72, age (mean = 20), first-year nursing students. No other demographic data was reported	Non-virtual environment, nursing program	High-fidelity simulator cardiac auscultation training	Traditional teaching method in an occupational skills laboratory	Knowledge assessment form for cardiac auscultation, Skills evaluation form for cardiac auscultation, State Anxiety Inventory		High-fidelity simulator teaching method was significantly more effective in improving the students’ knowledge and skills in cardiac auscultation and decreased their anxiety level compared to the traditional teaching method
Xiong et al. [45] 2017, China	To assess the effectiveness of a mixed media education intervention to enhance nursing students’ knowledge, attitude, and compliance with Standard precautions (SPs)	No data	N = 84 nursing students in final year of education, age (mean = 20.3 years), 84 female	No information about virtual environment provided, nursing program	An online learning group using Tencent QQ (a communication program) Biweekly mixed media education sessions, consisting of lectures, videos, role play, and feedback with 15–20 min of individual online supervision and feedback sessions following each class	The control group learned the same material as the intervention group through self-directed readings	Pre- and post-test assessments of knowledge, attitudes, and compliance with the Standard Precautions Questionnaire	Small sample size	At 6-week follow-up, performance on the knowledge, attitudes and compliance were significantly improved in the intervention group compared to the control group The intervention group performed significantly better in the hand hygiene standard
*Midwifery students*									
Amanak [51], 2020, Turkey	To compare low-fidelity simulation and hybrid simulation techniques for teaching how to perform intramuscular injections	Principal investigator	N = 73 first-year midwifery students, age (mean = 18.7 years). No other demographic data was reported	No information about virtual environment provided, midwifery program	Intramuscular injection through hybrid simulation	Intramuscular injection using the model routinely employed in the midwifery program	General Self-Efficacy Scale (GSES), State Trait Anxiety Inventory (STAI), and Guide to Performing Intramuscular Injections into the Ventrogluteal Site (GPIIVS)		At postintervention, the students in the hybrid simulation group had significantly higher self-efficacy, better performance of the injection and lower anxiety levels compared to the control group

### Method for the synthesis of results

A quality evaluation of the studies was not performed as this scoping review aimed to explore the literature rather than to analyse any intervention effects. The results were reported descriptively, and the study characteristics are presented in Table 2. The results were synthetized in the following topics: teaching and learning environments, digital teaching methods, characteristics of the digital teaching activities, outcome measures or results of the digital teaching interventions.

## Results

### Selection of sources of evidence

**Fig. 1:**
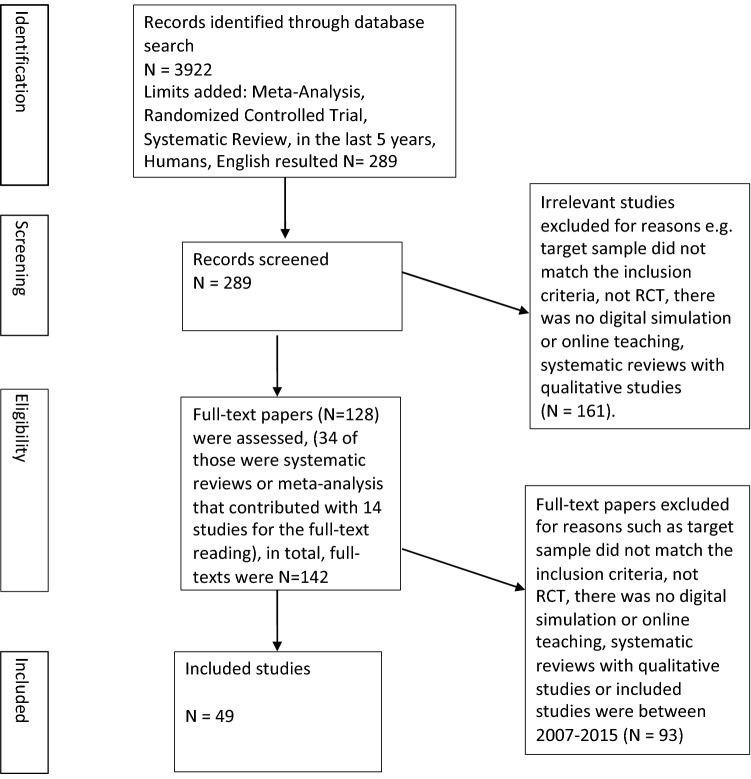
PRISMA chart for the study selection process

Five databases were included for the search. Figure 1 shows the PRISMA chart detailing the number of studies and the study selection process. Reasons for the rejection of the abstracts (N = 161) and full text papers (N = 93) varied (see Fig. 1). There were no relevant studies on higher education for the social professions. A total of 49 studies were included in the scoping review. See Table 2 for details on these studies.

### Characteristics of the studies

The included studies were from Europe [24–43]; Asia [44–55]; Oceania [56, 57]; and North America [58–72].

The target groups included medical students (n = 30) [24–35, 37, 38, 40, 41, 48, 49, 56–59, 61, 62, 64–66, 69–71], nursing students (n = 16) [36, 42, 43, 45, 47, 50, 52–55, 60, 63, 67, 68, 72], midwifery students (n = 1) [51], and dental students (n = 3) [39, 44, 46].

In total, 4092 students (mean age 23.5 years) participated in the included studies. The study population included students from the following programs: n = 207 dentistry; n = 2764 medicine; n = 1121 and nursing and midwifery. Students from the 1st to 6th educational years were included, comprising bachelor’s and master’s levels. The selected studies included a total of 2821 bachelor’s students and 104 master’s students. The year of study for the remaining 1167 students was unknown.

The study characteristics are presented in detail in Table 2.

### Synthesis of the results

#### Teaching and learning environments

Eighteen studies [24, 30, 34, 36–39, 44, 46, 49, 53, 54, 56, 58, 60, 62, 64, 69] were developed in a face-to-face format and were then transformed to digital format but did not use a virtual learning environment (i.e., there was no platform for content management, lesson planning, engagement, administration, communication). In 21 studies [25, 26, 29, 31, 33, 35, 40, 42, 45, 47, 48, 50–52, 55, 57, 63, 65, 67, 71, 72], the authors did not provide information about the learning environment, and in five studies [27, 41, 43, 59, 61, 68], the authors only mentioned that an e-learning/online learning system or a learning management system was used. One study reported that a shareable weblink was used but offered no specific information [32]. Regarding the remaining four studies, four different virtual learning environments were used: Basic Burns Management e-learning tool [66], Education in Dermatology [41], the VBLaST-PC system for fundamentals of laparoscopic surgery [70], and the CASUS—a case-based multimedia learning and authoring system for undergraduate, postgraduate and continuing education [28]. Thus, the studies showed substantial variation in the learning environment, making it difficult to draw conclusions of any kind. See Table 2 for more details.

#### Digital teaching methods

Teaching methods comprised three main categories: blended (also labelled as hybrid), e-learning or other online-based, and digital simulation-based. Many studies have used digital simulation-based teaching and learning methods. Seven studies used blended teaching methods [29, 32, 46, 47, 61, 65, 72], 15 studies used e-learning/online methods [27, 28, 30, 31, 33, 40, 41, 43–45, 56, 60, 66, 68, 71], and 20 studies [24, 25, 34, 36–38, 42, 48–55, 63, 64, 67, 69, 70] used digital simulation-based teaching and learning methods. Blended teaching methods and digital simulation-based teaching and learning methods were employed together in 5 studies [26, 39, 57–59], and e-learning/online and digital simulation-based teaching and learning methods were implemented together in 2 studies [35, 62]. The abovementioned teaching methods included many tools, e.g., different types of virtual simulator models, digital scenarios, digital patients, and environments that could have been web-based or not. Four studies [28, 34, 46, 70] used virtual reality (VR)/Augmented Reality (AR), 22 studies [24, 31, 36–39, 42, 43, 49–54, 57, 58, 60, 63, 64, 67, 69, 72] used virtual simulator (VS), four studies [30, 55, 56, 61] used video, and four studies [32, 33, 41, 62] used web-based technological tools. Some studies also utilized more than one tool. Two studies [25, 26] applied VR/AR and VS, 9 studies [27, 40, 44, 45, 47, 59, 65, 66, 68] implemented videos and web-based tools, two studies [35, 71] used VS and web-based tools, one study [48] used VS and video, and one study [29] implemented VR/AR, video and web-based technological tools together. See Table 2 for more details.

Communication in practical skills was taught with, e.g., an electronic Clinical Reasoning Educational Simulation Tool [33], Mpathic-VR for advanced communication skills [69] and using telehealth [70]. Mixed reality guidance systems [29], mobile platforms [31], and games [36, 37], among others, were implemented. Some studies used a single digital teaching method, while others used more than one. Thus, although the teaching and learning methods could be divided into only three categories, the tools used in teaching were nearly as numerous as the interventions in the included studies, which is beneficial for prompting teachers to try new methods. However, the use of many different tools can also pose a barrier since it is impossible to discuss the effects of any given tool. See Table 2 for more details.

#### Characteristics of the digital teaching activities

The analysis of the characteristics of digital technology applications is presented according to target groups defined by their profession. Two main approaches characterize all the studies, which dealt either with practical skills as technical skills taught through any digital method or communication in practical skills as nontechnical skills for practice taught through any digital method. Five studies aimed to develop both technical and nontechnical skills.

Dental students practised merely to develop practical technical skills related to the very core of their profession, for example cavity preparations [39] and the creation of mandibular molars [46]. However, in one study [44], the communication in the interaction between students and mentors was assessed.

Nursing and midwifery students practised specifically to develop practical technical skills, which were addressed in all 17 nursing and midwifery studies. These skills were most often described as various skills needed to perform clinical procedures, e.g., nasogastric tube placement [63], intramuscular injections [51], intravenous catheter placement [52], and cardiac auscultation [54]. Safety issues, such as standard safety precautions [45] and operating room fire safety issues [72], were also considered. Practical nontechnical skills training for nursing and midwifery students was reported in two studies. These skills were related to communicating with deteriorating patients before procedures [43] and communication in clinical decision-making [50].

The characteristics of medical students’ activities and practices were mainly technical, such as diagnosis of ear pathologies [62], paediatric basic life support [30], clinical examination [30], resuscitation [37], laparoscopy [26], infant laparoscopic fundoplication [57], suturing and tying skills [57], surgical skills in robotic surgery [48], and emergency ultrasound [27]. Five studies described the pursuit of medical students’ practical nontechnical skills. These included e.g., advanced communication intercultural communication [69], management of cognitive load [28], care communication with patients suffering from intellectual and developmental disabilities [61], and clinical reasoning [33]. See Table 2 for more details.

#### Outcome measures

The outcome measures were categorized by applying the four levels developed by George Miller [73] for the assessment of clinical skills, competence, and performance: 1. Knows (knowledge); 2. Knows How (competence); 3. Shows How (performance); 4. Does (Action). The first two levels, Knows and Knows How, were not easy to distinguish from each other; thus, these were considered as one level in our summary. We also added an Other category, which included e.g., beliefs, attitudes and values.

Twenty-four studies [27–30, 32, 33, 35, 41, 42, 44–47, 50, 52–55, 60, 62, 65, 66, 71, 72] reported outcomes on Miller’s [73] levels 1–2, and 42 studies [24–29, 31, 33, 34, 36–40, 43, 44, 46–54, 56–72] reported outcomes on Miller’s level 3. Several studies (n = 17) [27–29, 33, 44, 46, 47, 50, 52–54, 60, 62, 65, 66, 71, 72] reported outcomes combining Miller’s levels 1–3. No studies reported outcomes on Miller’s level 4, i.e., Does, which concerns the performance of practical skills outside the digital teaching context independently in a clinical context. Outcomes that were categorized as “other” were reported in 19 studies [29, 32, 33, 35, 36, 42, 45–47, 51, 52, 54, 59–61, 63, 64, 66, 71].

The outcomes on the Knows/Knows How level were measured with, e.g., the Knowledge with standard precautions questionnaire [45], Knowledge questions on performing urinary catheterization [47], and the Knowledge assessment form in the management of preeclampsia [60]. The outcomes on Shows How level measured e.g., suturing skills [57], or the Creighton Simulation Evaluation Instrument (CSEI) for rater-observation to measure competence in patient scenarios [68]. The Other category included outcome measures regarding students’ satisfaction with the digital pedagogical methods [71], feasibility and acceptability of the teaching method [33], level of confidence in practical skills [32], teaching preference and learning experiences [29], attitudes towards digital teaching method [63], anxiety [64], motivation [47], compliance [45], and learning self-efficacy [42]. See Table 2 for more details.

#### Results of the digital teaching interventions

The results showed that the use of digital technologies in practical skills teaching and learning has a wide range of outcomes. A total of 16 studies [24, 28, 34, 42, 44, 46, 50–52, 54, 59, 62, 64–66, 70] showed that students in the intervention group significantly improved their practical skills compared to the control group of students who studied with traditional teaching methods. There was also a significant trend in the development of knowledge, as 11 studies [31, 32, 35, 40–42, 45, 50, 54, 63, 68] showed that the use of digital technologies, which make learning pathways more flexible, also helped students to acquire and strengthen the knowledge that underpins the acquisition and development of practical skills. Four studies [43, 56, 61, 69] showed the development of students’ practical nontechnical skills (communication skills). Furthermore, three studies [52, 64, 71] showed that students’ learning motivation increased, five studies [32, 35, 47, 52, 64] showed that students' confidence in their abilities was strengthened, and three studies [51, 54, 64] showed that the use of technologies in a safe study environment reduced students’ anxiety about manipulations that must be performed in the working environment in the future. Four studies [36, 42, 47, 52] also showed an increased level of satisfaction with the learning processes that used innovative methods.

Despite these promising results, 17 studies [25–27, 29, 33, 36–39, 48, 49, 55, 57, 58, 60, 67, 72] showed the same level of practical skill development in the intervention group using digital technologies and in the control group using traditional teaching methods. In three studies [37, 48, 67], the results of the use of different technologies in the intervention and control groups, such as online courses vs. digital games, were compared, but the learning outcomes were the same in both groups. It should be clarified that in situations where the intervention and control groups showed similar results in the context of skill development, however, there was nevertheless an increase in the intervention group, for example, in the learning motivation or confidence. Two studies [30, 53] showed that students in the control group using traditional teaching methods had a higher increase in their practical skills compared to the intervention group using digital technologies. See Table 2 for more details.

## Discussion

The findings of this study showed that digital learning environments (e.g., digital teaching and learning platforms) were not used in nearly half of the studies even though the digital teaching method was applied in all included studies. Teaching methods were blended, e-learning or other online-based, and digital simulation-based. The teaching and learning environments, methods, resources, and characteristics of the activities varied considerably making summary difficult and hindering the conclusions of the effects of any specific digital teaching tool. Half of the studies measured outcomes at the knowledge level and not at the performance level. One-third of the studies showed a significant improvement in practical skills in the intervention group in comparison to the control conditions. However, one-third showed no differences in practical skills between groups, even though confidence and motivation in practical skills were increased when compared to the control group.

Digital teaching and learning environments, methods, and resources varied greatly in the included studies, which have also been reported by others [74]. Additionally, many used digital teaching methods without a digital learning platform as support. The great variation makes it impossible to comment on recommendations for future use for digital practical skills teaching and learning in health care education programs. A well-functioning learning environment [14], especially the digital learning environment, is important in education for digital teaching and learning of practical skills and should be a focus when developing new digital practical teaching methods.

The translation of face-to-face interventions to digital versions does not work well [75]. The empirical experience during the COVID-19 pandemic has also clearly illustrated that the face-to-face format is not directly transferable to the remote learning format and that the alignment of technology use with learning objectives and learning outcomes is not always obvious and does not necessarily lead to better learning quality. In an integrative review Turnbull et al. [75] identified several challenges when translating face-to-face teaching to remote teaching in higher education, e.g., the digital competency of teachers and students and integration of learning tools in a classroom interacting in “real-time” and tools that are used by students at their own pace and in interactions with each other and their teachers over longer periods of time. Teachers’ lack of digital skills when adapting face-to-face education to digital format has also been reported by Kenzig [76]. Turnbull et al. [75] further identified successful strategies for translation from face-to-face to digital format, e.g., supporting teachers’ and students’ digital competency and broadening the face-to-face course with components of blended learning. Using digital learning and teaching in higher education can eliminate geographical proximity and increase the diversity of the student population [77]. Additionally, this characteristic implies that the students can be educated wherever they are for at least part of their program.

Our scoping review showed that the studies’ teaching methods were blended, e-learning or other online-based, or digital simulation-based. It emerged that the digital simulation teaching seems to support the students’ active learning of skills and competencies in authentic environments. However, the importance of the teacher´s role in supporting the student's commitment and dialogue was not reflected as clearly, despite its importance, especially in blended and distance learning, as recommended within the framework of a student-centred, and competency-based approach to learning to facilitate student engagement in the learning process [17].

The knowledge level of measuring outcome of the teaching intervention was seen in half of the studies. McCutcheon et al. [74] found that 7 of 19 included studies on teaching clinical skills in undergraduate nursing education had some type of performance outcome measure, mostly a checklist of clinical skills needed for a task. They also reported that knowledge, self-efficacy and user satisfaction were measured as was reported in our review. Practical skills must be measured at the knowledge level but to measure outcomes at the performance level should in future research be planned in the study protocol.

Students’ general satisfaction with the used digital environments, methods and resources used for practical skills teaching and learning in our review has also been shown in previous descriptive studies [12, 13], implying that the students might be more prepared to accept digital methods in practical teaching than the teachers may believe. Furthermore, a systematic review of digital learning effectiveness in the physiotherapy education context [8] revealed that 19 of 21 studies showed significant differences in knowledge acquisition for digital interventions in comparison to control conditions. However, it is possible that the effects were based on only the acquisition of theoretical knowledge rather than practical skills learning, which was delivered on campus [8]. Evidence of the effectiveness of digital teaching in practical skills is conflicting, one systematic review showed positive results for blended teaching [78], and another showed no effects [79] of digitally assisted instruction for the task of physical examination. In our review, which was focused only on digital practical skills teaching and learning, one-third of the included studies showed positive results in knowledge acquisition. However, there were no differences in many of the studies comparing digital teaching methods to traditional classroom educational methods. Obtaining comparable results with digital teaching methods or using the face-to-face method could also be interpreted positively, i.e., digital teaching was not inferior to the face-to-face method. McCutcheon et al. [74] came to a similar conclusion regarding studies on teaching clinical skills to undergraduate nurses. The comparable results should not be underestimated considering the extreme challenge faced by higher education due to COVID-19 in recent years.

We need to carefully examine our existing methods in digital practical skills teaching and learning to introduce more effective and user-friendly new methods. Future studies could include case-study design investigations that explore concrete cases and descriptions of how lecturers have developed students' practical skills in different study formats (blended and online learning, etc.). The COVID-19 period has brought about the rapid development of various digital learning and teaching solutions, but the workload of lecturers during the transition from face-to-face to online formats has been very substantial, and there has been no opportunity to scientifically record events from which to draw evidence-based conclusions and publish them in peer-reviewed journals. Therefore, a case-study design would be a more appropriate format for delivering additional results to the research community and developing discussions about students’ skills development in blended and online learning settings.

Our scoping review has some strengths. A rigorous and comprehensive search strategy was developed during several meetings with the author group. This search strategy, while implemented for a scoping review, would also have been appropriate for a systematic review. The search itself was conducted by library staff who were specially educated for these purposes. The evaluation of the included studies and writing of the results were performed by the author group, thus avoiding a bias that could have emerged if only one or two individuals had done the same work. Furthermore, the number of included studies was high, and all the studies offered higher evidence levels as judged by their design, i.e., randomized controlled design. Thus, the internal validity of our results could be considered high with one exception: We did not evaluate the quality of the included studies, which could have been low, thereby causing problems with internal validity.

There are some additional limitations as well. We limited the inclusion period to studies published no more than 5 years before the search and published in English. No grey literature was included. The 5-year limitation was decided in an authors’ meeting due to the presumably fast development of methods in the digital teaching and learning of practical skills. Had the 5-year limit produced only a few results, we would have increased the limit to 10 years. Nonetheless, the more recently developed methods are more relevant to today’s teachers than the older methods. We included only RCT-designed studies because of the high scientific level of design characteristics and their quality. However, a true RCT design in the education intervention context can be problematic in several ways. For example, the blinding of the participants is very difficult in the education context, intervention contamination between groups occurs, and it is seldom possible to have a control condition without any education intervention since the curriculum must be followed. These all are thus limitations of this scoping review. The English language requirement and grey literature exclusion may have influenced the results. However, we were able to include 49 studies, giving our scoping review quite high reliability. Using numerous filters could be a problem, but we consider that in our case, the limitations caused by filters are not highly problematic.

The studies included in our scoping review represent a global sample, meaning that the results could possibly be generalized to health and social education programs in the dentistry, medicine, nursing, and midwifery fields. However, the studies were carried out in a certain local context, implying that there are probably several context-related variables that can affect the results and thus possibly decrease the generalizability of this review. Furthermore, unfortunately, no studies on other health and social education programs were found during our 5-year study period.

The theoretical knowledge can be easier and more effectively taught and learned through digital resources than practical skills teaching. Thus, the development of digital teaching methods for practical skills must first identify the real problem areas for this kind of teaching rather than beginning with an existing model and content developed for campus-based teaching. We should learn to include the end-users in the development of digital teaching methods, much as they are included in the development of new mobile and other digital interventions in health care research today. In our case, the end-users are the students and teachers, not the patients or clients.

### Conclusions

The teaching and learning methods comprised three categories, blended, e-learning/online and digital simulation-based, but the used digital tools used varied greatly, as did the learning environments, making it difficult to draw conclusions. The use of digital technology, in the learning and teaching process can contribute to the development of not only of students' skills but also their knowledge, motivation, and attitudes. The authors of the study would like to highlight that the pedagogical factor of how technology is used is decisive. Furthermore, the results suggest that there are positive implications for using digital practical skills teaching and learning methods, but the digital methods may be at their best when used alongside with more traditional face-to-face methods. The development of new digital methods for teaching and learning practical skills also requires engaging students and teachers, not only researchers.

## Data Availability

The included studies are available by contacting the first author. The scoping review was registered in the Open Science Framework, OSF registry (Registration https://doi.org/10.17605/OSF.IO/BVP3M).
